# Liver cholesterol matters

**DOI:** 10.18632/aging.104208

**Published:** 2020-10-26

**Authors:** Bishuang Cai, Xiaobo Wang

**Affiliations:** 1 Department of Medicine, Icahn School of Medicine at Mount Sinai, New York, NY 10029, USA; 2 Department of Medicine, Columbia University Irving Medical Center, New York, NY 10032, USA

**Keywords:** NASH, cholesterol, TAZ, HIPPO, liver fibrosis

Nonalcoholic steatohepatitis (NASH) progresses from nonalcoholic fatty liver disease (NAFLD), which is common in the elderly and is an important predictor of liver failure and hepatocellular carcinoma (HCC). NASH also has a robust and multifaceted relationship with aging-related diseases such as metabolic syndrome, cardiovascular diseases, and diabetes. Due to older people’s activity limitations, pharmacological treatment is essential, but an incomplete understanding of NASH pathophysiology limits drug development. Cholesterol (low-density lipoprotein) has been identified as a cardiovascular disease risk factor for a few decades. Recently, liver free cholesterol was proven as a NASH inducer [1].

**Figure 1:**
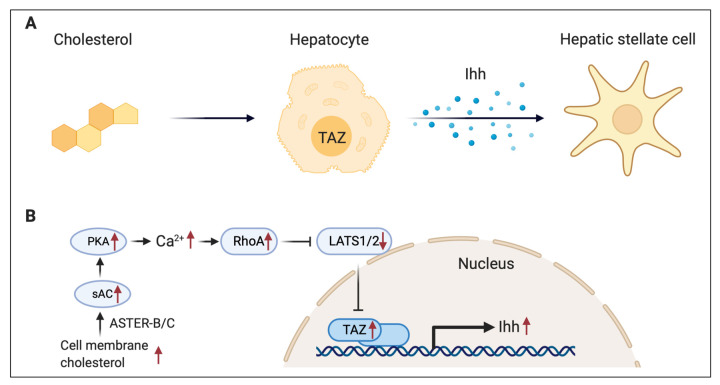
**Summary scheme of the cholesterol-mediated hepatocyte and stellate cell crosstalk pathway in NASH.** **(A)** In NASH, elevated cholesterol upregulates TAZ in hepatocytes, and TAZ activates hepatic stellate cells by inducing Ihh. **(B)** In hepatocyte, ASTER-B/C-mediated plasma membrane cholesterol internalization activates PKA and calcium signaling through soluble adenylyl cyclase (sAC). The resulting increase in RhoA activity inhibits LATS1/2 and promotes TAZ nucleus translocation. As a transcription factor, high level of TAZ enhances Ihh mRNA expression.

In human NASH liver, there are increases in both cholesterol and TAZ (also known as WWTR1, transcriptional coactivator with PDZ-binding motif) protein, yet the molecular mechanism linking cholesterol to TAZ upregulation in NASH remains undetermined. TAZ is one of the transcriptional regulators in the Hippo pathway that play roles in development, organ size and homeostasis, and carcinogenesis. TAZ is negatively regulated by upstream signaling: MST1/2 kinases activate LATS1/2 kinases to phosphorylate TAZ, suppressing its activity by proteasomal degradation. Dephosphorylated TAZ translocates into the nucleus, where it interacts with TEAD to activate specific target gene expression. A dedicated study by Wang et al. [1] uncovered a pathway that elucidated that excess hepatic cholesterol increases TAZ through calcium-Hippo-mediated signaling transduction. First, they found that liver cholesterol is elevated in both human and mouse NASH livers. The upregulated cholesterol increases liver and hepatocyte TAZ expression *in vivo* and *in vitro* due to inhibited degradation through cholesterol-blocked Hippo activity. They also described a signaling pathway that includes cholesterol transporter AsterB/C, soluble adenylyl cyclase (sAC), PKA, and IP3R. Via this pathway cholesterol is transported into cytoplasm and mediates downstream calcium signaling to induce RhoA activity and suppresses Hippo kinase-Lats1/2, leading to increased TAZ and YAP (another TAZ homologue in the Hippo pathway) protein at the post-transcriptional level. The elevated TAZ triggers the fibrosis and induces NASH [2] (Figure 1). Interestingly, the YAP function may diverge even it was regulated in the same way as TAZ. Wang et al. demonstrate a new role for cholesterol trafficking in regulating cAMP, calcium, and Hippo transcription factors. These findings provide a pathophysiological mechanism linking elevated liver cholesterol to NASH and raise the possibility of new therapeutic targets. Liver cholesterol is not like plasma cholesterol and is difficult to measure due to limited biopsy. Therefore, new tools and methods are required to screen this risk factor in the future.

In another issue of Cell Metabolism, Wang et al. [2] investigated the role of elevated TAZ in fibrotic liver in NASH. They demonstrated that there was a marked elevation of TAZ expression in three different mouse models, as well as in human NASH livers. Hepatocyte-specific knockout TAZ diminished liver inflammation and fibrosis without changing steatosis. Conversely, TAZ overexpression sufficiently enhanced histologic features of NASH and related gene expression. Interestingly, hepatocyte TAZ was not induced in the liver injury model, and TAZ silencing did not affect CCl4-induced fibrosis, which suggests that elevated TAZ may not be a general feature of liver injury but is related explicitly to NASH pathology. The mechanism of TAZ-induced fibrosis in NASH was determined *in vivo* and *in vitro*: TAZ in hepatocytes induces the synthesis and secretion of Indian hedgehog (Ihh), and Ihh activates hepatic stellate cells to promote fibrosis. The study further introduced an important new mechanism to understand the role of cholesterol-induced TAZ in hepatocytes in animal models with corroborating evidence in human NASH, and suggested TAZ as a plausible therapeutic target in NASH. Although there are safety concerns about targeting TAZ specifically in hepatocytes, lowering TAZ properly in chronic disease should be feasible since TAZ expression in the healthy liver is very low or undetectable compared with NASH liver.

After the first hepatocyte-targeted siRNA was approved for clinical use, hepatocyte-specific protein, TAZ, was targeted by the stabilized GalNAc-siRNA in NASH [3]. The results demonstrated that TAZ siRNA suppresses steatosis progression to NASH and reverses liver fibrosis and inflammation in a dietary mouse model of NASH. Steatohepatitic HCC is strongly associated with NASH. Another interesting avenue to explore would be whether blocking the TAZ pathway prevents NASH-associated HCC development.

In summary, the findings of Wang et al. [1] have identified a new pathway into the complex milieu of NASH pathogenesis and have led to new pharmaceutical molecule development. Hippo pathway signaling is dysregulated in aging, and the role of cholesterol-TAZ signaling in the elderly population in NASH needs to be investigated.
